# Effects of platelet-rich plasma on the activity of human menstrual blood-derived stromal cells in vitro

**DOI:** 10.1186/s13287-018-0795-3

**Published:** 2018-02-26

**Authors:** Siwen Zhang, Pingping Li, Zhengwei Yuan, Jichun Tan

**Affiliations:** 1 Reproductive medicine Center, Obstetrics and Gynecology Department, Shengjing Hospital affiliated to China Medical University, No. 39 Huaxiang Road, Tiexi District, Shenyang, 110022 China; 2 Key Laboratory of Health Ministry for Congenital Malformation, Shengjing Hospital affiliated to China Medical University, No. 7, Economic Development Zone, Benxi, 117004 China

**Keywords:** Menstrual blood-derived stromal cells, Platelet-rich plasma, Endometrial receptivity, MenSCs

## Abstract

**Background:**

Human menstrual blood-derived stromal cells (MenSCs) are highly proliferative and show multiple differentiation capacity. The convenience and non-invasiveness make MenSC a novel cell source for regenerative medicine applications. Platelet-rich plasma (PRP) contains abundant growth factors which are beneficial to wound healing. However, the influence of PRP on MenSCs remains elusive. Here, we evaluated the role of PRP in MenSCs proliferation and assessed the effects of PRP on endometrial receptivity regulation in vitro.

**Methods:**

MenSCs cultured with 10% activated PRP were compared with those cultured with 10% fetal bovine serum (FBS). Differences in cell proliferation, differentiation, and endometrial receptivity-related gene expression were evaluated.

**Results:**

Notably, 10% activated PRP significantly promoted MenSCs proliferation and adipogenic/osteogenic differentiation while suppressing apoptosis. Expression of the mesenchymal stem cells (MSCs) marker CD105 and the perivascular markers SUSD2 and CD146 were elevated after PRP treatment. Moreover, short-term PRP stimulation activated the phosphorylation of Akt and signal transducer and activator of transcription 3 (STAT3) pathways, upregulated expression of *FoxO1*, *LIF,* and *IL1-β,* and downregulated *IL-6*.

**Conclusions:**

In summary, PRP could promote MenSC proliferation, markedly accelerate cell stemness, and evaluate MenSC functions by enhancing the expression of angiogenesis and endometrial receptivity markers, suggesting its potential use as a promising supplement for MenSCs in endometrial regenerative medicine. Our results provide a theoretical basis for the clinical application of co-transplantation of PRP combined with MenSCs.

**Electronic supplementary material:**

The online version of this article (10.1186/s13287-018-0795-3) contains supplementary material, which is available to authorized users.

## Background

Menstrual blood-derived stromal cells (MenSCs) are isolated human endometrial stem cells (hENSCs) obtained during menstrual shedding [1]. MenSCs are located in the basalis and functionalis of the endometrium and are thought to play a key role in endometrial regeneration [2]. The perivascular markers CD146, platelet-derived growth factor receptor (PDGFR)-β, and sushi domain-containing 2 (SUSD2) can be used as specific markers for isolation of MenSCs directly from the endometrial shedding mixture by flow cytometry [3]. Moreover, cells with high expression of SUSD2 or co-expression of SUSD2 and CD146 generate more colony-forming units (CFUs) than the CD146^+^PDGFR-β^+^ subpopulation in the proliferative endometrium, suggesting that these stem cells have a role in functionalis growth [4]. In addition to the basic characteristics of mesenchymal stem cells (MSCs), i.e., self-renewal, clonogenicity, and multipotency, MenSCs exhibited higher extraction efficiency and longer passaging capacity [5]. Moreover, MenSCs show improved proliferation capability with a shorter population doubling time of 20 h [6], which is twofold faster than that of bone marrow-derived mesenchymal stromal cells (BM-MSCs).

In previous studies, neither tumor growth nor toxicity was detected after MenSCs treatment in animal models. Moreover, the injection of MenSCs resulted in substantial inhibition of tumor development [7]. Recent reports have shown the potential clinical applications of MenSCs in the treatment of type 1 diabetes mellitus [6], stroke [8], ovarian failure [9], Asherman’s syndrome [10], and epithelial ovarian cancer [11]. In our previous study, we transferred autologous MenSCs to seven infertile women who were diagnosed with severe Asherman’s syndrome and demonstrated that the endometrial thickness of all patients was significantly increased, including in one patient who had an ongoing pregnancy. Our results suggest that MenSCs transplantation may be a promising option for endometrial regeneration and improvement of endometrial receptivity improvement [10].

Current concerns with stem cell-based therapy focus on the surgical procedures involved in sample collection. Compared with other MSCs, MenSCs are free of ethical concerns and can be harvested easily and noninvasively from menstrual blood, therefore, making them attractive for applications in regenerative medicine [7, 12]. Generally, fetal bovine serum (FBS) is the most common medium supplement for therapeutic stem cell culture in vitro. However, the use of FBS-based culture is associated with the risk of infectious disease and immunological reaction during cell transplantation treatment [13].

Human platelet-rich plasma (PRP) is an artificial blood-derived product with the significant advantage of enabling autologous transplantation. In small volumes of plasma, the density of platelets in PRP is 5–10 times to that in whole blood. Platelets are commonly concentrated from the peripheral blood via two or three steps using different centrifugal forces or via mechanical collection [14]. The active ingredient, platelets, are rich in growth factors (GFs) and cytokines, including vascular endothelial growth factor (VEGF), transforming growth factor β-1 (TGFβ-1), platelet-derived growth factor (PDGF), epidermal growth factor (EGF), hepatocyte growth factor (HGF), and insulin-like growth factor (IGF-1) [15]. These therapeutic molecules are released after platelet activation and are beneficial for cell proliferation and wound healing [16]. Moreover, by serving as a cellular scaffold, PRP boosts the potency of transplanted cells used in stem cell-based therapies [17]. In vitro, PRP has been clearly demonstrated to increase cell biological functions. Adult cells, such as epithelial cells, chondrocytes and osteoblasts as well as multipotent cells, such as adipose tissue-derived stem cells (ADSCs), BM-MSCs, and tendon stem cells have been reported to show enhanced proliferation after PRP treatment [18–20]. Besides promotion of proliferation, differentiation, and recruitment are also observed, suggesting potential beneficial effects in tissue regeneration [21]. Moreover, human platelet products have been shown to be more effective in induction of osteogenic lineage differentiation of MenSCs compared with FBS-containing culture medium [22, 23]. In addition to basic nutritional support, PRP has also been shown to be a promising substitution for FBS in cell culture because of its self-collectivity, low cost and low immunogenicity [23].

In this study, we aimed to evaluate the effects of PRP on the regulatory effects of MenSCs on biological and endometrial function in vitro. Our results provide important insights into the mechanisms through which PRP affects the activity of MenSCs in vitro and the value of PRP in autologous transplantation for improvement of endometrial receptivity.

## Methods

### MenSCs isolation and culture

Six menstrual blood donors were diagnosed without reproductive system diseases, aged 25–35 years old. All donors gave consent and all procedures were approved by the Ethics Committee of Shengjing Hospital affiliated to China Medical University (2017PS330K).

MenSCs were collected and individually cultured according to our previous report [10]. Briefly, after vagina disinfection, 5 ml samples of menstrual blood were collected on day 2 of menses with a 20-ml injection syringe. Then the menstrual blood was transferred onto Ficoll (Sigma-Aldrich, St. Louis, MO, USA), fractionated at a density-gradient centrifugation according to the manufacturer’s instructions. The central cell layer with purified mononuclear cells were washed and cultured in 25-cm^2^ tissue culture bottles (NEST) with Chang’s medium: Dulbecco’s modified Eagle medium: nutrient mixture F-12 (Ham’s) (1:1, HyClone, Logan, UT, USA) with 10% FBS (Gibco, Waltham, MA, USA) and 1% penicillin-streptomycin (Sigma-Aldrich). In a humidified incubator, cells were grown under 37 °C in an atmosphere containing 5% CO_2_. After 24 h the medium was changed and the attached cells were washed by phosphate-buffered saline (PBS). The culture medium was changed every 3 days until adherent cells reach 80–90% confluence, then the cells were passaged by 0.25% trypsin (Sigma-Aldrich).

### Platelet-rich plasma (PRP) preparation

Platelet-rich plasma was extracted from apheresis platelets (a platelet-plasma mixture with a concentration of 12.5 × 10^11^/L, from a healthy female donor aged 30), acquired from the blood transfusion department of Shengjing Hospital. 20% CaCl_2_ (Sigma-Aldrich) plus 1000 U/ml thrombin from bovine plasma (T8020, Solarbio, Beijing, China) was used for activation, added into the apheresis platelets at a volume ratio of 1:20. The mixture was incubated at 37 °C for 1 h and at 4 °C for 12 h. For collection of activated PRP, the gel-like mixture was centrifuged at 5000 rpm for 30 min at 4 °C. The supernatant was aspirated and filtered through 0.22-μm filters and then aliquoted and stored at −80 °C, to avoid repeated freezing and thawing.

### Cell proliferation assay

Cell-counting kit 8 (CCK-8) cell proliferation assay was performed by cell counting kit-8 (CCK8; Dojindo, Kumamoto, Japan) for 7 days. We set the culture medium with gradient concentrations of activated PRP (20%, 10%, 5%, 2.5%) as experimental groups, 10% FBS as the control group and serum-free medium as the blank control group. P4 MenSCs were seeded onto 96-well plates at approximately 2 × 10^3^ cells per well with Chang’s medium. After 24 h, the original medium was exchanged as grouped and refreshed every 3 days. Every day at an indicated time, wells containing cells were washed once by PBS then 100 μl DMEM and 10 μl CCK8 was added to cells, incubated at 37 °C for 2.5 h. Optical density (OD) was measured by 450 nm absorbance. A MTS (Promega, Beijing, China) assay (for 7 days) was performed as a verification of CCK8 assay. 20 μl MTS was added to cells and incubated for 2.5 h. The results are shown in Additional file 1: Data 1.

### EdU proliferation assay

EdU assay was performed to certificate the proliferation promotion effect of PRP. We assessed 10% PRP as the experiment group and 10% FBS as the control. P4 4 × 10^3^ MenSCs were plated onto 96-well plates per well. The medium was changed into serum-free medium overnight when the cells grew to a density of 40%. After that the cells were incubated by PRP or FBS for 24 and 48 h, then they were disposed as per the EdU manufacturer’s instructions (C00031, Riobio, Guangzhou, China). The immunoflurorescence was captured by Nikon Eclipse Ni (Nikon, Tokyo, Japan), the positive rate was analyzed by Image Pro Plus 6 (Media Cybernetics Rockville, MD, USA).

### Flow cytometry

P4 MenSCs were maintained in 100-mm culture dishes (BD Falcon, BD Biosciences, San Jose, CA, USA) for flow cytometry. Briefly, after being incubated in serum-free medium overnight to synchronize the cell cycle, cells were cultured with 10% PRP or 10% FBS for 24 h or 48 h. Then the cells were collected and tested.

We measured surface makers CD34, CD38, CD44, CD45, CD73, CD90, CD105, SSEA-4, CD146, and SUSD2 after 24-h incubation. 1 × 10^6^ cells were suspended for 100 μl in 1% BSA/PBS incubated with antibodies of CD34-FITC, CD38-PE, CD44-FITC, CD45-FITC, CD73-PE, CD90-PE, CD105-FITC, or SSEA-4-PE (BD, Franklin Lakes, NJ, USA). CD146-FITC and SUSD2-PE (Biolegend, San Diego, CA, USA) were co-incubated. For apoptosis analysis, 1 × 10^6^ cells were stained with Annexin V-FITC/PI kit following the manufacturer’s protocol (#88–8007, eBioscience, San Diego, CA, USA). Briefly, the cells were washed twice by PBS and suspended with 100 μl binding buffer, 5 μl Annexin V-FITC was added into each tube. After incubated in the dark for 15 min, the cells were washed then diluted to 200 μl with 5 μl propidium iodide (PI), and analyzed by flow cytometry. For cell cycle analysis, cells of each group were fixed with 70% ethanol-PBS at 4 °C for 24 h, then be washed and suspended with 300 μl PBS. The suspension was treated with 3 μl RNase A (10 mg/ml, Solarbio) and 3 μl PI, 10 mg/ml (Solarbio) and washed in water at 37 °C for 30 min. These cells were analyzed by fluorescence-activated cell sorting using a flow cytometer (BD FACSCalibur; BD Biosciences). The rates of apoptosis and phenotype were analyzed by CellQuest, cell cycle was quantified by ModFit LT for Mac v3.0 (BD Biosciences).

### Mesenchymal stem cell differentiation properties

To assess the influence of PRP on MenSCs differentiation, we cultured P4 MenSCs in adipogenic and osteogenic induction medium with 10% activated PRP or 10% FBS.

We seeded over 10^5^ MenSCs in 35-mm culture plates pre-incubated with Chang’s medium, setting PRP treatment as the experimental groups and FBS treatment as the control groups. When cell density reached 30%, the osteogenic induction began (ascorbic acid 2 μl/ml, beta-glycerophosphate sodium 10 μl/ml, dexamethasone 0.1 μl/ml) while the density reached 100% for adipogenic treatment (medium A: insulin 2 μl/ml, IBMX 1 μl/ml, rosiglitazone 1 μl/ml, dexamethasone 0.1 μl/ml; medium B: insulin 2 μl/ml). All the cells were cultured in induction medium (Cyagen, Guangzhou, China) for 3 weeks (six cycles) at 37 °C in 5% O_2_/5% CO_2_/90% N_2_, medium changed as protocol. Then the cells were fixed and specifically colored to detect the differentiation: Oil Red for adipogenesis and Alizarin Red for osteogenesis (Cyagen). Stained cells were examined and photographed using Nikon ECLIPSE Ni (Nikon).

### Immunofluorescence microscopy

P4 MenSCs were cultured on coverslips (14 mm, NEST) at 5 × 10^4^ cells/ml in 12-well plates. After overnight starvation, the medium was exchanged to 10% PRP or 10% FBS-DMEM/F12 incubated for 24 h. Then the coverslips were washed twice by PBS and fixed in 4% PFA, followed by protein block (SP-9001, ZSGB-BIO) for 30 min at 37 °C. Purified SUSD2 antibody (1:100, #327401, BioLegend), CD146 antibody (1:100, ab75769, Abcam, Cambridge, MA, USA), Vimentin antibody (1:100, D21H3, CST), cytokeratin 18 (CK18) antibody (1:100, ab181597, Abcam) diluted in PBS were immunostained overnight at 4 °C. Next day, all the coverslips were incubated with secondary antibodies (1:500, Cy3-labled goat anti-mouse IgG, FITC-labeled goat anti-rabbit IgG, Cy3-labeled goat anti-rabbit IgG, Beyotime, Beijing, China) at room temperature for 2 h. DAPI (1:20, Beyotime) was used to visualize nuclei. Images were observed and captured using Nikon ECLIPSE Ni (Nikon), the fluorescence was analyzed by DOI/Area using Image Pro-Plus 6 (Media Cybernetics).

### Quantitative RT-PCR

P4 MenSCs were used to extract total RNA with RNAiso Plus (#9108, Takara, Tokyo, Japan), the expression of *FoxO1, HOXA10, LIF, CK18, IL1-β, IL6, RUNX2, and PPARγ2* was analyzed. Grouped as introduced previously, cells were conditionally cultured for 6 h or 24 h. Cells that were serum-starved overnight were set as control. The RNA was reverse transcribed into cDNA using PrimeScript RT Regent Kit (#RR047A, Takara) according to the manufacturer’s protocol following by the RT reaction that as 37 °C for 15 min, 85 °C for 5 s and 4 °C. All the cDNA was stored at −20 °C. Quantitative PCR was performed using SYBR Premix Ex Taq ii (#RR820A, Takara). The primers used in this study are listed in Table 1. Quantitative RT-PCR was conducted at 95 °C for 30 s followed by 40 cycles at 95 °C for 5 s and 60 °C for 34 s and final extension at 60 °C for 15 s in 7500 software v2.0.6 (Life Technologies, Carlsbad, CA, USA). The relative levels of mRNA were normalized with GAPDH, gene expression was analyzed by 2^-ΔΔCt^.

**Table 1:** Quantitative polymerase chain reaction primer sequences

Name	Sequence (5′-3′)
*FoxO1*	F: TACGAGTGGATGGTCAAGAGC
	R: TGAACTTGCTGTGTAGGGACA
*HOXA10*	F: CTGAGGTCAATGGTGCAAAGGA
	R: TTTGCCAACCTGCATGTCCA
*LIF*	F: CCAACAGCAAGACGAGGATG
	R: GATGAAGCAGGAAGGAGAAGG
*CK18*	F: ATCTTGGTGATGCCTTGGAC
	R: CTCAGAACTTTGGTGTCATTGG
*IL-1β*	F: TGGCAATGAGGATGACTTGT
	R: TGGTGGTCGGAGATTCGTA
*IL-6*	F: TTCGGTCCAGTTGCCTTCT
	R: GGTGAGTGGCTGTCTGTGTG
*RUNX2*	F: GCAGCAGCAGCAGCAGGAG
	R: GCACCGAGCACAGGAAGTTGG
*PPARγ2*	F: AGAACAGATCCAGTGGTTGCAGATTAC
	R: CAGACACGACATTCAATTGCCATGAG
*GAPDH*	F: CAGGAGGCATTGCTGATGAT
	R: GAAGGCTGGGGCTCATTT

### Western blot

The treatment and groups of cells were the same as RT-PCR treatment. Cells were scraped off the bottles with 250 μl radio immunoprecipitation assay (RIPA) buffer combined protease inhibitor (PMSF) (P0013B, ST506, Beyotime). The lysates were collected by centrifugation at 14,000 rpm for 20 min at 4 °C. Total protein was qualified by BCA kit (P0010S, Beyotime), samples were diluted to 2 μg/μl. Protein samples were separated by 8% sodium dodecyl sulfate-polyacrylamide gel electrophoresis (Beyotime) and 0.45 μm PVDF membranes (EMD Millipore, Billerica, MA, USA) were used for transferred, 5% non-fat powder milk dissolved in TBST was used for blocking. Thereafter, all membranes were incubated with primer antibodies listed below for 12 h at 4 °C (Akt #4691, 1:1000, Stat3 #4904, 1:1000, Phosopho-Akt (Thr308) #13,038, 1:1000, Phosopho-Akt (Ser473) #4060, 1:1000, Phosopho-Stat3 (Tyr705) #9145, 1:1000, FoxO1 #2880, 1:1000, all from Cell Signaling Technology, Danvers, MA, USA; LIF antibody #abs 120615 from Absin, 1:300; IL1-β antibody #16806–1-AP, 1:500, IL6 antibody #21865–1-AP,1:500 from (Proteintech, Chicago, IL, USA). Subsequently, the membranes were incubated on the following day with secondary antibody (peroxidase-conjugated affinipure goat anti-rabbit IgG, ZSGB-BIO, Beijing, China) for 1 h at room temperature. Thereafter, the blots were added ECL (#1862420, #1862421, Thermo Fisher Scientific, Waltham, MA, USA) and visualized by C300. The gray densitometric was analyzed using Image J (National Institutes of Health, Bethesda, MD, USA).

### Statistics

Descriptive statistics and statistical analysis were carried out using GraphPad Prism 5 (San Diego, CA, USA). Results are presented as mean ± SEM. One-way ANOVA test was used in CCK8 and MTS statistical analysis and two-tailed unpaired *t* test was used in other experiments. *P < 0.05, **P < 0.01, ***P < 0.001 were considered as statistical significant.

## Results

### PRP promoted MenSCs proliferation while protecting cells from apoptosis

**Fig. 1:**
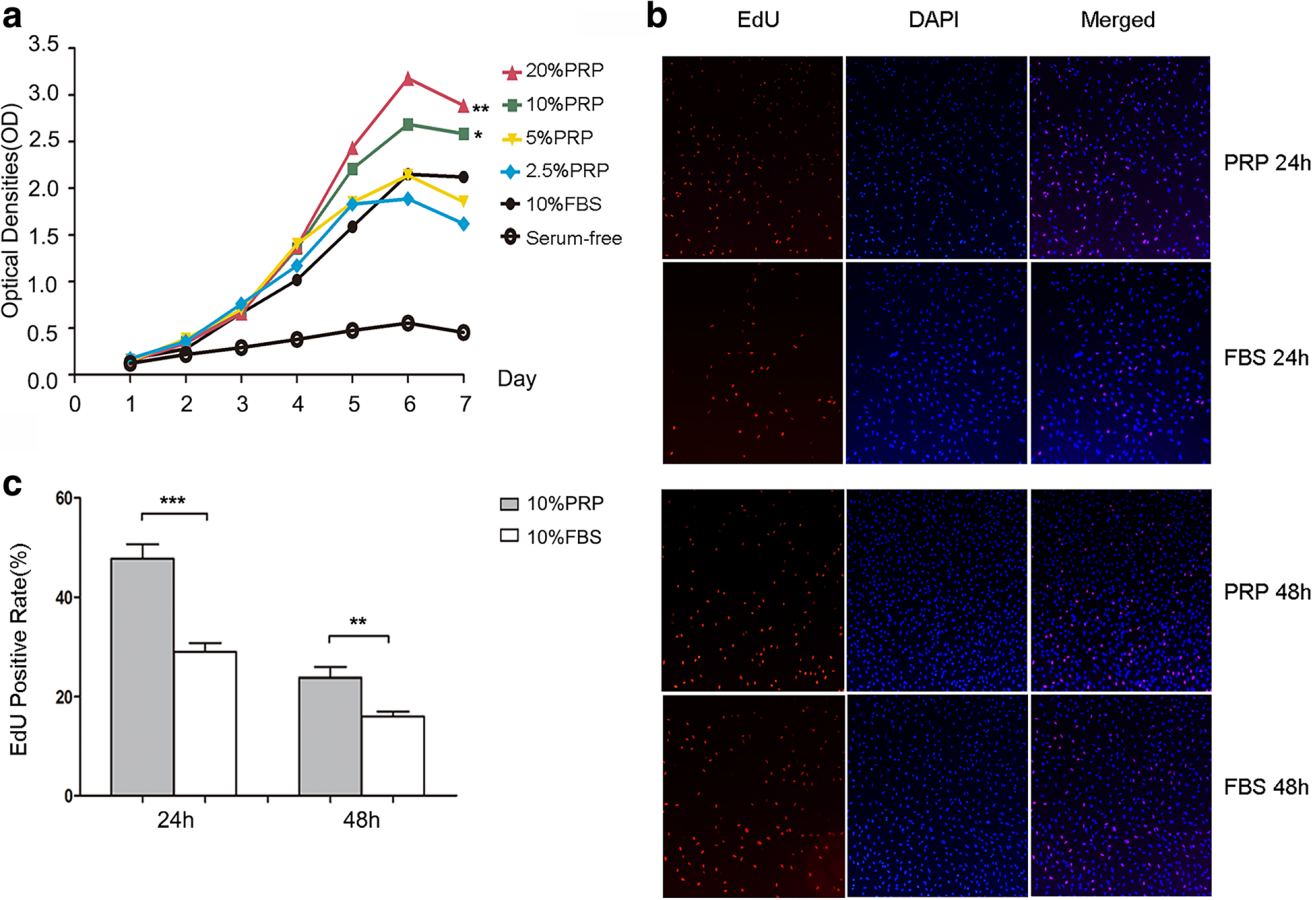
PRP promoted MenSCs proliferation. **a** CCK8 assay detected proliferation of P4 MenSCs cultured with different concentrations of activated PRP or 10% FBS (*n* = 3). **b** Immunofluorescence analysis of EdU+ MenSCs under stimulation of 10% PRP and 10% FBS for 24 h and 48 h (*red*: DNA replicating, *blue*: nuclei, *n* = 6). **c** Statistical analysis of EdU+ cell rate of each group. MenSCs cultured with 10% PRP showed higher proliferation rate for both 24 h and 48 h (*P* < 0.01). CCK8 was analyzed by one-way ANOVA test. Data was mean ± SEM, *P < 0.05, **P < 0.01, ***P < 0.001 for two-tailed *t* test

First, we examined the effects of PRP on MenSCs proliferation using cell counting kit-8 (CCK8) assays (*n* = 3). As shown in Fig. 1a, compared with the serum-free group, activated PRP significantly increased the number of viable cells and showed positive concentration-dependent effects. During the first 3 days, optical density (OD) values in all serum-in groups were nearly identical. Compared with 10% FBS, groups with PRP showed increased cell proliferation beginning on day 4, and this effect persisted for days. OD values of 10% and 20% activated PRP were significantly higher than those of the 10% FBS group. Accordingly, all further experiments were conducted with 10% PRP. The OD values of several groups decreased slightly on day 7, probably because the cells were confluent. MTS assays showed the same trend as CCK8 results (Additional file 1: Data 1).

Furthermore, we assessed cell proliferation using 5-ethynyl-2′-hydeoxyuridine (EdU, *n* = 6), a thymidine analog that can permeate into DNA molecules at the S stage instead of thymidine when diluted in culture medium. As shown in Fig. 1b and c, more EdU-positive cells were found in the PRP groups than in the FBS groups (*P* = 0.0003 and 0.0022 for 24 and 48 h, respectively). Generally, the positive rate was much higher after 24 h of culture than after 48 h of culture in both groups. These results indicated that PRP acted positively on DNA replication in MenSCs, suggesting that PRP may elicit stronger effects after a shorter incubation.

**Fig. 2:**
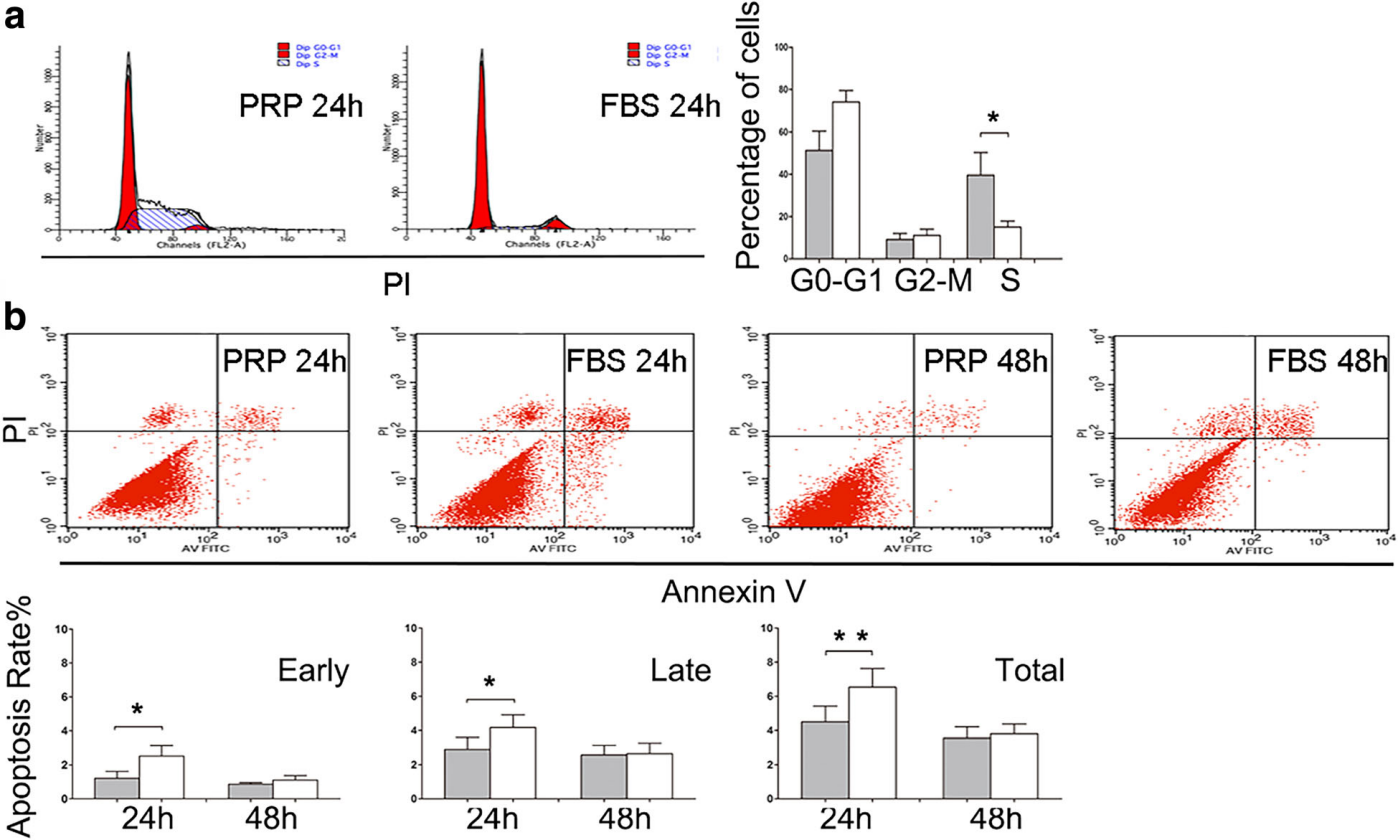
PRP affected cell cycle and apoptosis of MenSCs. **a** Flow cytometry analysis of cell cycle (n = 6) after treatment with 10% PRP or 10% FBS for 24 h. The percentage of cells in S stage was significantly higher in MenSCs of PRP group, demonstrating a DNA synthesis promotion. **b** Flow cytometry detection of cell apoptosis by Annexin V/PI staining. P4 MenSCs (n = 6) were treated for 24 h or 48 h then were tested. Early, late, and total apoptosis declined after PRP treatment for 24 h. Data was mean ± SEM, *P < 0.05, **P < 0.01, ***P < 0.001 for two-tailed *t* test

Subsequently, to explore the mechanisms involved in PRP-dependent MenSC proliferation, we detected the cell cycle of both groups using flow cytometry (*n* = 6). As shown in Fig. 2a, after treatment with PRP for 24 h, there was a significant increase in the number of cells in the S phase (*P* = 0.029) compared with those in the FBS group. There were no differences between the two groups for cells in the G_0_/G_1_ phase or G_2_/M phase. Moreover, as shown in Fig. 2b, early (Annexin V^+^ PI^−^), late (Annexin V^+^PI^+^) and total apoptosis (Annexin V^+^) in P4 MenSCs were significantly inhibited after PRP treatment for 24 h (*P* = 0.0376, *P* = 0.0178 and *P* = 0.0079, respectively, *n* = 6) as compared with the control. However, at 48 h, the differences were not significant. These results indicated that PRP promoted MenSC proliferation by upregulation of DNA replication and downregulation of cell apoptosis.

### PRP elevated the pluripotency of MenSCs

**Fig. 3:**
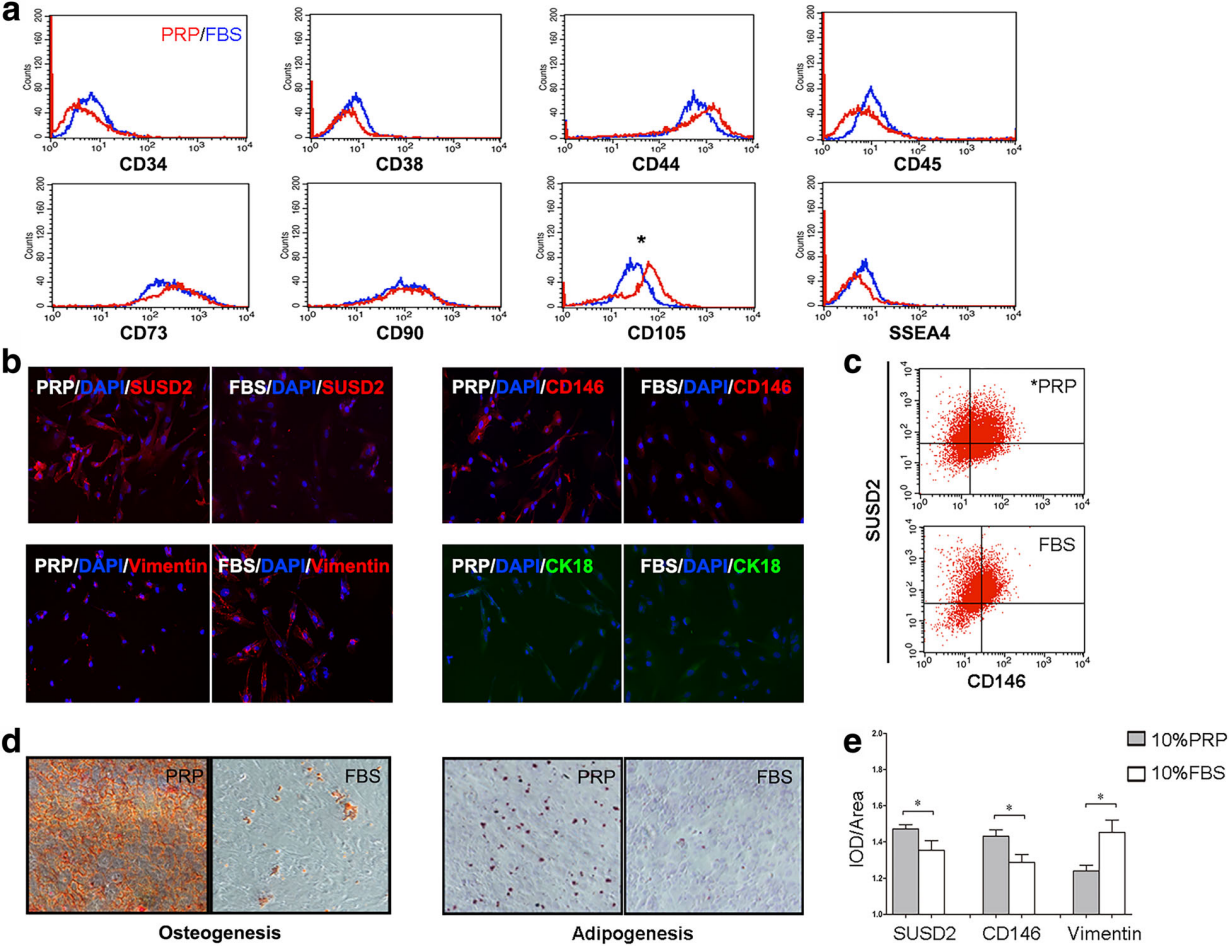
Pluripotency of MenSCs incubated with PRP. **a** Flow cytometry analysis of MSC surface markers CD34, CD38, CD44, CD45, CD73, CD90, CD105, and SSEA4 (n = 6). *Red lines* for PRP, *blue lines* for FBS. CD105 was significantly increased after PRP treatment for 24 h, *P* < 0.05. **b** Immunofluorescence assays of SUSD2, CD146, Vimentin, and CK18 (*red* or *green*), DAPI was used to stain nuclei (*blue*) (n = 6). **c** Perivascular markers SUSD2 and CD146 co-expression assessed by flow cytometry (*P* < 0.05, *n* = 4). **d** Osteogenic and adipogenic differentiation of MenSCs treated with 10% PRP or 10% FBS for 21 days (×100), staining by Alizarin Red for calcium nodules and Oil Red for lipid droplets after fixation (n = 3). **e** Fluorescence intensity analysis of SUSD2, CD146, and Vimentin in B. Data was mean ± SEM, *P < 0.05, **P < 0.01, ***P < 0.001 for two-tailed *t* test

Flow cytometry was applied to evaluate changes in stemness markers of MenSCs following treatment with PRP (*n* = 6). As shown in Fig. 3a, there were no significant differences in the expression of CD34, CD38, CD44, CD45, CD73, CD90, and SSEA-4 after PRP treatment for 24 h. However, the expression of CD105, which participated in angiogenesis, was upregulated following PRP treatment (*P* = 0.0381). PRP treatment also increased the co-expression of perivascular selection markers CD146 and SUSD2 (*P* = 0.0493, *n* = 4; Fig. 3c).

Consistent with these findings, immunofluorescence also showed increased SUSD2 and CD146 expression in MenSCs in the PRP group (*n* = 6). As shown in Fig. 3b, the intensities of SUSD2 and CD146 were significantly increased in the PRP-treated group (*P* = 0.0489 and 0.0238, respectively). The expression of vimentin, a stromal cell marker, and cytokeratin 18 (CK18), an epithelial cell marker, were further analyzed (n = 6). Reduced expression of vimentin (*P* = 0.0117) was found in the PRP group, suggesting the role of PRP in maintaining the pluripotency of MenSCs. In contrast, the intensity of CK18 was weak in both groups and showed no significant difference.

To determine whether PRP affected the differentiation capacity of MenSCs, we treated P4 MenSCs with osteogenic or adipogenic induction medium for about 21 days (six cycles according to the protocol; Cyagen) with 10% PRP or 10% FBS (*n* = 3). After treatment with PRP plus osteogenic induction medium, MenSCs exhibited strong Alizarin Red-stained calcium nodules, with greater amounts than the control (Fig. 3d, left). For adipogenic differentiation, cells treated with PRP plus induction medium showed greater Oil Red O staining (Fig. 3d, right), indicating more lipid droplet formation than cells in the FBS group.

### PRP regulated the expression of endometrial receptivity- and inflammation-related genes and proteins in MenSCs

**Fig. 4:**
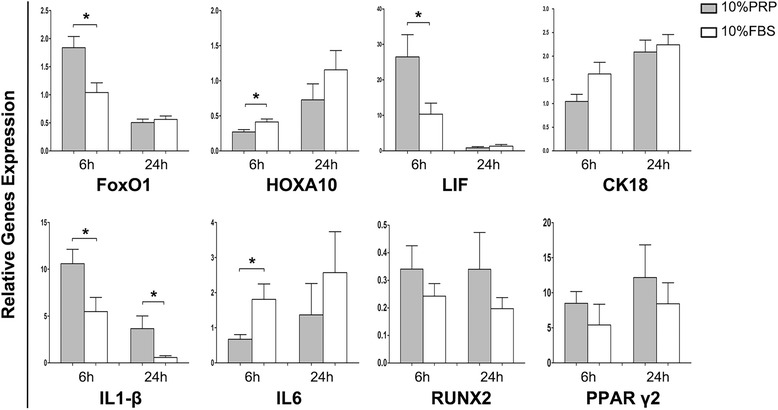
Quantitative RT-PCR analysis of receptivity- and inflammation-related genes. Gene expression analyzed by RT-PCR. P4 MenSCs (*n* = 6) cultured with 10% PRP (*gray*) or 10% FBS (*white*) for 6 h and 24 h. RT-PCR analysis of *FoxO1*, *HOXA10*, *LIF*, *CK18*, *IL1-β*, *IL6, RUNX2,* and *PPARγ2*. *GAPDH* was used for mRNA standard, fold changes were measured by 2^-ΔΔCT^. Data was mean ± SEM, *P < 0.05, **P < 0.01, ***P < 0.001 for two-tailed *t* test

To ensure the efficiency of PRP-assisted MenSCs in the treatment of endometrial damage, real-time polymerase chain reaction was used to evaluate the expression of endometrial receptivity- and inflammation-related genes in MenSCs cultured with PRP for 6 or 24 h (*n* = 6). As shown in Fig. 4, *FoxO1* expression was higher in PRP-treated cells than in FBS-treated cells at 6 h (*P* = 0.0105). Similarly, *LIF* gene expression was significantly increased in PRP-treated cells compared with that in FBS-treated cells at 6 h (*P* = 0.044), while weak expression was detected in both groups at 24 h. In contrast, the expression of *HOXA10* was significantly decreased following PRP treatment at both 6 and 24 h, compared with that after FBS treatment (*P* = 0.02 for 6 h). After PRP treatment, *IL1-β* expression was upregulated at 6 h and 24 h (*P* = 0.0429 and 0.0497) and *IL6* was downregulated (*P* = 0.0336) at 6 h. *CK18* gene expression was upregulated, albeit not significantly, at both 6 h and 24 h. No significant differences in *RUNX2* or *PPARγ2* (osteogenic and adipogenic genes) were detected at both 6 h and 24 h.

**Fig. 5:**
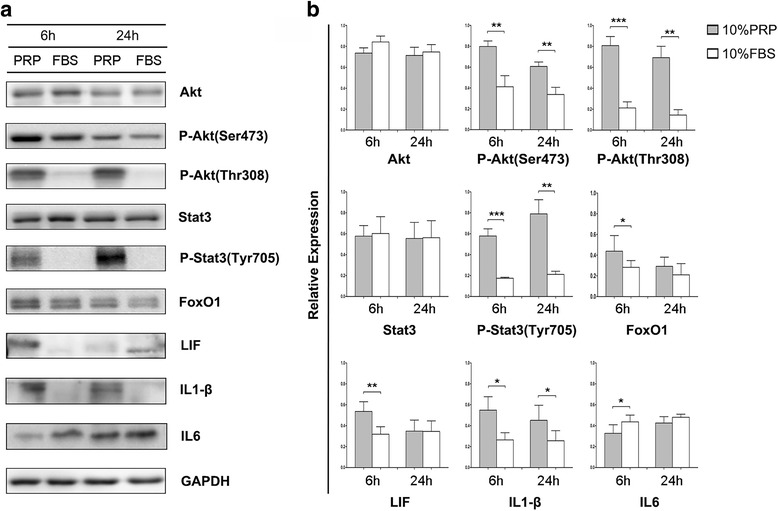
Western blot analysis of signal pathways. **a** P4 MenSCs were cultured with 10% PRP or 10% FBS for 6 h and 24 h, and cell lysates were immunoblotted (n = 6). PRP activated phosphorylation of Akt and Stat3 pathways and enhanced FoxO1, LIF, and IL1-β expression, and decreased IL6 expression. **b** Gray level analysis of western blots. Abbreviation of P-represented phosphorylation. Data was mean ± SEM, *P < 0.05, **P < 0.01, ***P < 0.001 for two-tailed *t* test

Western-blot for FoxO1 showed the same trends as observed for mRNA expression (*P* = 0.0422 at 6 h) and LIF was increased significantly at 6 h for PRP incubation (*P* = 0.0096). After PRP treatment, IL1-β levels were increased at 6 h and 24 h (*P* = 0.0141 and 0.031) and IL6 level was decreased at 6 h (*P* = 0.0286) (Fig. 5a and b).

These data demonstrated that PRP could stimulate the expression of endometrial receptivity- and inflammation-related genes and proteins.

### PRP activated Akt and signal transducer and activator of transcription 3 (STAT3) pathways in MenSCs

We then analyzed the expression levels of proteins in the Akt and STAT3 pathways in MenSCs after PRP treatment for 6 or 24 h (*n* = 6). As shown in Fig. 5a and b, phospho-Akt (Thr308) and phospho-STAT3 (Tyr305) levels were significantly increased after PRP induction (*P* = 0.0002 and 0.001 for phospho-Akt (Thr308) at 6 and 24 h, respectively; *P* = 0.0003 and 0.0032 for phospho-STAT3 (Tyr305) at 6 and 24 h, respectively). Levels of phospho-Akt (Ser473) were also enhanced in the PRP group (*P* = 0.0084 and 0.0079 at 6 and 24 h, respectively). The expression of total Akt was reduced in the PRP group, which indicated that the increased phospho-Akt could be at least partly explained by increased phosphorylation of AKT.

## Discussion

MenSCs are a type of MSCs that exhibit all generalized MSC characteristics and have several advantages over MSCs. In contrast to BM-MSCs or ADSCs, the process of harvesting MenSCs is completely non-invasive. In addition, MenSCs have a shorter population doubling time and do not show changes in traits according to donor age during early passages (up to passage 10) [24]. As a novel type of graft in regenerative medicine, MenSCs have been safely used in the treatment of several diseases based on its low immunogenicity [25].

In this study, we evaluated the effects of human PRP, a promising autologous product, on MenSCs. Several studies [26] have clearly demonstrated that activated PRP promotes the proliferation of many MSCs lines in a concentration-dependent manner in vitro. Consistent with these findings, our study revealed that higher concentrations of activated PRP had stronger effects for MenSCs culture duration. According to the results of CCK8 assays, we concluded that 10% activated PRP was the optimal concentration for MenSCs proliferation, similar to the results of a previous study [27], in which 10% PRP was found to have the greatest effects on the proliferation of tendon progenitor cells. In this study, activated PRP increased the ratio of cells in the S phase and inhibited apoptosis, probably via activation of the Akt and IL-6/STAT3 pathways. These results showed that the growth factors released by PRP accelerated DNA replication in MenSCs and promoted cell proliferation.

PRP is a potential growth factor supplement that has been suggested to act as a catalyst and to accelerate MSC differentiation without affecting cellular structure and biology affects. Abundant GFs in PRP such as PDGF, IGF, and TGF-β have been reported to have stimulatory effects on stem cell differentiation [28]. According to a previous study on BM-MSC, *RUNX2,* and *PPAR* γ*2*, the master genes expression for osteogenic and adipogenic genes were slightly upregulated after treatment with PRP releasate for 5 days [21]. Similar results were also detected in ADSC osteogenic differentiation after PRP treatment for 21 days [23]. General mineralization markers such as Alizarin Red and alkaline phosphatase were also increased as a result of PRP addition, suggesting that osteoblast differentiation and matrix mineralization may be modulated by *RUNX2* expression [29]. Similar results were also detected in ADSCs after PRP treatment for 21 days [19]. However, according to a study by Amable, no significant differences in adipogenic gene expression were detected in differentiated BM-MSCs with PRP incubation for 21 days, although the stimulatory effects of PRP on adipogenic differentiation were obvious [30]. The results of the present study showed that 10% activated PRP promoted osteogenesis and adipogenesis in MenSCs (21 days). However, no significant differences in related gene expression were detected at 6 h or 24 h, probably because of the short incubation time. Further studies are needed to elucidate the mechanisms through which PRP affects MenSC differentiation.

In this study, the PRP we used was extracted from apheresis platelets, similar to clinical autologous pure PRP (P-PRP) separated from leukocytes and pro-inflammatory cytokines. P-PRP has been shown to be more suitable for bone regeneration and acute tendon injury healing [31], indicating an anti-inflammatory effect. A previous study demonstrated that PRP provides the immunoregulatory effects of downregulating crucial inflammatory cytokines, such as interleukin IL-6 and IL-8. *IL6* is a pro-inflammatory cytokine and participated in the implantation window. It is essential to form an anti-inflammatory state following implantation in order to prevent fetal rejection [32]. In this study, it was demonstrated that the expression of IL-6 was downregulated in MenSCs treated with PRP compared with that in cells treated with FBS, suggesting an anti-inflammatory effect in endometrium. Another pro-inflammatory cytokine, IL1-β, was upregulated after PRP incubation. IL1-β is also known to be increased in the mid-secretory phase of human endometrium, which is essential for embryo implantation [33]. Moreover, increased IL1-β may be due to platelet activation [34] and it decreased immediately within 24 h. Weak release of pro-inflammatory cytokines following platelet activation has been suggested to occur after stimulation of cell proliferation, resulting in activation of tissue remodeling in vivo [35].

In vivo, hENSCs positive for SUSD2 and CD146 were found to be highly proliferative and to undergo self-renewal in the endometrium. These cells are located around the vessels in the functionalis and basalis and participate in endometrial regeneration [4]. Moreover, SUSD2^+^ hENSCs in the decidua could exhibit paracrine signaling through production of cytokines and secretomes, suggesting that they may participate in the regulation of the endometrial microenvironment [36]. In our study, the levels of SUSD2, CD146, and CD105 were increased after PRP treatment for 24 h, suggesting the role of PRP in promoting MenSC engraftment in endometrial vascular regeneration in vivo. These results were consistent with previous studies showing that PRP-treated BMSCs could improve vascularization in bone regeneration [37, 38].

Moreover, we found that in the PRP-treated group, the expression levels of endometrial receptivity-related genes, including *FoxO1*, *LIF*, and *IL1-β*, were upregulated, suggesting that PRP functioned to improve endometrial function. We found that levels of *FoxO1* and *LIF* gene expression were increased by 1.73- and 2.68-fold, respectively, in the 6 h PRP-treated group compared with that in the 6 h FBS-treated group. *LIF* is one of the most important genes affecting endometrial receptivity and is weakly expressed in the endometrium of patients with unexplained infertility, suggesting an important role in blastocyst implantation [39]. *FoxO1* is a novel transcriptional factor involved in decidualization of endometrial stromal cells, functionally required for binding of progesterone [40]. Moreover, another previous report also demonstrated that *FoxO1* is associated with vascular homeostasis in the endometrium and a mediator of the AKT pathway, suggesting an essential role in vascular and embryonic development [41]. Similar results were reported in several studies of bone and cartilage regeneration, in which PRP engraftment resulted in good outcomes and promoted the expression of particular functional genes, such as bone morphogenetic protein-2 (*BMP2*) and *SOX9* [42]. However, *HOXA10*, another implantation window-related gene [43], was downregulated by PRP treatment. The mechanisms involved in this phenomenon are unclear. Thus, further studies are needed to expand on this basic in vitro study and fully elucidate the mechanisms involved in this process.

In 2012, Darzi also studied the effects of human platelets on MenSCs in vitro. He found that MenSCs are less capable of osteogenetic differentiation than BMSCs, and substitution of FBS with human platelet releasate could equalize these effects. Therefore, he suggested that human platelet releasate could be used as an osteogenic accelerator in MenSCs culture, making it a better substitute for BMSCs for bone tissue engineering [22]. In the present study, we investigated whether PRP regulated endometrial receptivity by affecting MenSCs. Our center is currently carrying out a clinical study on MenSCs transplantation as treatment of thin endometrium or intrauterine adhesions. According to our data, MenSCs transplantation can effectively increase thickness of endometrium [10], however, the pregnancy outcome is not ideal. We are committed to finding a safe and effective additive to improve the efficiency of MenSCs transplantation in curing uterine infertility. An animal study is currently ongoing to detect whether PRP affects MenSCs to improve the efficacy of transplantation in the treatment of uterine damage in vivo. We expect that autologous PRP combined with stem cell-based therapy may have excellent therapeutic effects on organic infertility and other diseases.

## Conclusions

Based on their advantages of improved proliferation and non-invasiveness, MenSCs are regarded as a promising stem cell resource for regenerative medicine applications. This study showed that activated PRP could act as an excellent stimulator for MenSCs. PRP promoted MenSCs proliferation and differentiation, and elevated the expressions of SUSD2, CD146, and CD105. In addition, PRP upregulated the endometrial function-related targets *FoxO1*, *LIF*, and *IL1-β,* and downregulated the inflammation-related target *IL-6* probably via phosphorylation-dependent activation of Akt and STAT3 pathways. This study is the first to indicate that PRP may regulate endometrial receptivity through effects on MenSCs. Thus, PRP may be a beneficial component in the culture of MenSCs used for endometrial regeneration therapy.

## Additional file

Additional file 1: Data 1 MTS assay for cell proliferation detection. MTS assay detected proliferation of P4 MenSCs cultured with different concentrations of activated PRP or 10% FBS (*n* = 3). The data was analyzed by one-way ANOVA test, *P < 0.05, **P < 0.01. (TIFF 804 kb)

## Abbreviations

ADSC: Adipose tissue-derived stromal cell
BM-MSC: Bone marrow-derived mesenchymal stromal cell
BMP2: Bone morphogenetic protein-2
CCK8: Cell counting kit-8
CFU: Colony-forming unit
CK18: Cytokeratin 18
EGF: Epidermal growth factor
FBS: Fetal bovine serum
HDP: Human platelet derivative
hENSC: Human endometrial stem cell
HGF: Hepatocyte growth factor
IGF-1: Insulin-like growth factor
MenSC: Menstrual blood-derived stromal cell
MSC: Mesenchymal stem cell
OD: Optical density
PBS: phosphate-buffered saline
PDGF: Platelet-derived growth factor
PDGFR: Platelet-derived growth factor receptor
PRP: Platelet-rich plasma
RIPA: Radio immunoprecipitation assay
SUSD2: Sushi domain-containing 2
TGFβ-1: Transforming growth factor β-1
VEGF: Vascular endothelial growth factor
