# Supplementary figures and images for: Effects of platelet-rich plasma on the activity of human menstrual blood-derived stromal cells in vitro
Source: Stem Cell Res Ther. 2018 Feb 26;9:48. doi: 10.1186/s13287-018-0795-3 (PMC6389087; doi:10.1186/s13287-018-0795-3)

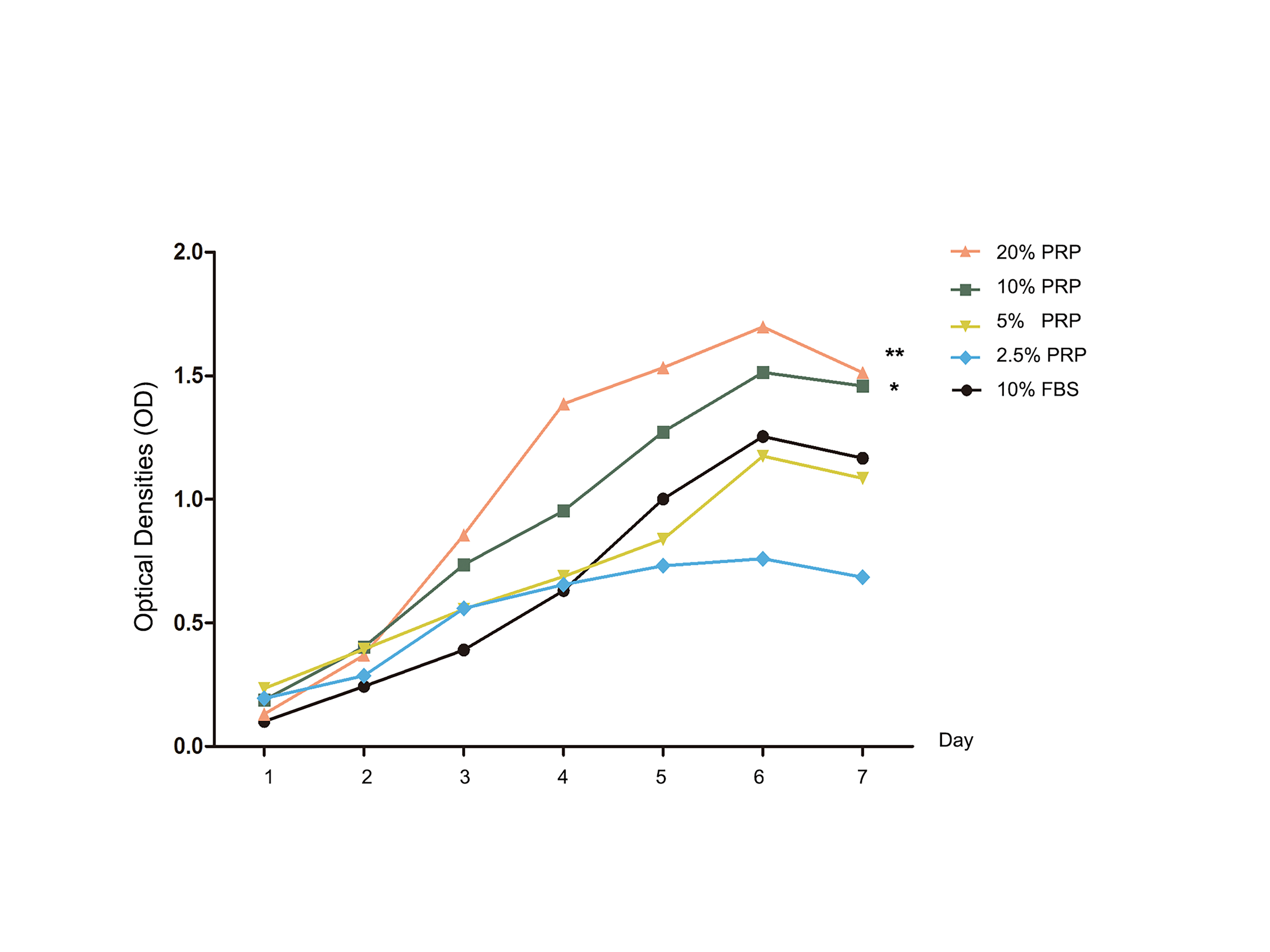

Supplement: Supplementary file 1 — Data 1 MTS assay for cell proliferation detection. MTS assay detected proliferation of P4 MenSCs cultured with different concentrations of activated PRP or 10% FBS (n = 3). The data was analyzed by one-way ANOVA test, *P < 0.05, **P < 0.01. (TIFF 804 kb) [file 13287_2018_795_MOESM1_ESM.tif]
